# Bibliometric and visual analysis in the field of traditional Chinese medicine in cancer from 2002 to 2022

**DOI:** 10.3389/fphar.2023.1164425

**Published:** 2023-07-04

**Authors:** Facheng Bai, Zhenguang Huang, Jun Luo, Yue Qiu, Shuwen Huang, Chenglong Huang, Taotao Liu, Hongliang Zhang, Dandan Wang

**Affiliations:** ^1^ Pharmacy Department, The Second Affiliated Hospital of Guangxi Medical University, Nanning, Guangxi, China; ^2^ Pharmacy Department, The First Affiliated Hospital of Guangxi Medical University, Nanning, Guangxi, China

**Keywords:** bibliometric analysis, traditional Chinese medicine, cancer, network pharmacology, metabonomic

## Abstract

**Objective:** Traditional Chinese medicine (TCM) has been used as a complementary treatment for cancer patients, but there has been no quantitative comprehensive analysis of TCM’s efficacy. The purpose of this paper is to explore the current status and hotspots of TCM in cancer research from 2002 to 2022 and to provide a reference for future research.

**Methods:** We retrieved articles published between 2002 and 2022 from the Web of Science database and analyzed them using R software, VOSviewer, and CiteSpace software.

**Results:** A total of 7,129 articles were included in this study. The publication rate of TCM cancer research increased steadily from 2002 to 2022, with a rapid increase from 2010 to 2021. China was the country with the most published articles, followed by the United States, Republic of Korea, Germany, and Japan. China was also the country with the most international collaborations, and China Medical University and Shanghai University of Traditional Chinese Medicine were the most representative cooperation centers. The Journal of Ethnopharmacology was the most published and cited journal. Apoptosis, expression, *in vitro*, activation, and other related keywords were commonly used in these articles. Breast cancer, colorectal cancer, gastric cancer, liver cancer, and lung cancer were the most studied cancer types in TCM research. Pathway-related apoptosis, anti-inflammation, and oxidative stress were the hotspots and trends of TCM’s anti-cancer mechanism. Metabolomics combined with network pharmacology was the main research method.

**Conclusion:** Traditional Chinese medicine as an anti-cancer drug has received increasing attention from researchers worldwide, and it is expected to be a hotspot for developing new anti-cancer drugs in the future. Our study provides a comprehensive analysis of the current status and hotspots of TCM cancer research, which could serve as a valuable reference for future studies.

## 1 Introduction

Cancer is a chronic disease that significantly affects human health and is the leading cause of death. It is also a significant obstacle to extending life expectancy (Fidler et al., 2018; Mattiuzzi and Lippi, 2020; Sung et al., 2021). In 2020, it was estimated that there were 19.3 million new cancer cases and nearly 10 million cancer deaths worldwide. By 2040, the global cancer burden is expected to reach 28.4 million, an increase of 47% over 2020 (Sung et al., 2021). At present, cancer treatment mainly comprises surgery (Katz et al., 2022), chemotherapy (Knezevic and Clarke, 2020), targeted therapy (Lee et al., 2018), radiotherapy (Koukourakis and Koukourakis, 2023), immunotherapy (Guo et al., 2019), and other methods. Among them, chemotherapy is the primary method of cancer treatment. However, despite the continuous progress in chemotherapeutic drugs, the effect of chemotherapy has been limited due to drug resistance and recurrence, which has resulted in reduced patient survival rates (Almeida et al., 2019; Assaraf et al., 2019; Vasan et al., 2019). Cancer treatment should aim to prolong the survival of patients while improving their quality of life (Cazzaniga et al., 2019). Therefore, cancer prevention and treatment remain a significant challenge in the world, particularly in low- and middle-income countries.

Traditional Chinese medical science, with a history of thousands of years, is a unique diagnosis and treatment method and one of the most important components of complementary and alternative medicine. Traditional Chinese medicine (TCM) is the most important part of complementary and alternative medicine (Yue et al., 2019; Li et al., 2021) and is widely used as health food and medicine to treat various diseases. By reading the classics of traditional Chinese medicine, such as Shen Nong Ben Cao Jing, Qian Jin Fang, Ben Cao Gang Mu, and others, valuable applications and principles of action for single and compound prescriptions have been found and are still in use today (Wang et al., 2014). TCM treatment is characterized by multi-component and multi-target individual regulation, which helps the body change from an abnormal state to a normal state (Wang et al., 2018). Chinese herbal medicine, which has attracted significant research interest, is the source of new drugs and drug precursors. Many modern drugs have been derived from herbal medicine and natural products, providing effective monomer chemicals for the development of medicine worldwide (Li and Vederas, 2009; Cragg and Newman, 2013).

For thousands of years, TCM has used thousands of single entities and complex prescriptions to prevent and treat human diseases. Early studies have shown that some herbs and natural compounds derived from them can effectively interfere with tumor progression, inhibit angiogenesis, and prevent metastasis (Chang, 2002; Lee et al., 2006; Sun et al., 2009). With the deepening of research, the pharmacological activities and mechanisms of natural compounds, herbal medicines, and complex prescriptions have been further revealed and demonstrated, leading to an increasing use of TCM as a clinically effective treatment for cancer (Hwang et al., 2012). In addition, many studies have shown that TCM can also overcome drug resistance by regulating the tumor microenvironment (Hwang et al., 2012), cell cycle (Xie et al., 2016), hypoxia (Hussain et al., 2018), tumor stem cells (Wei et al., 2014), autophagy (Sun et al., 2017), key signaling pathways such as the PI3K/Akt/mTOR pathway (Zhang et al., 2019), Hedgehog pathway (Sui et al., 2017), and others. Currently, active compounds extracted from herbs used to treat cancer have sparked significant interest. Because the development of cancer involves multiple genes and pathways, the use of TCM in cancer prevention and treatment may have more advantages than single-target drugs (Wang et al., 2014). Cancer treatment is multi-stage and complex, and TCM treatment has unique advantages and characteristics that differ from other types of anti-tumor treatments, which cannot be ignored. However, TCM still has a long way to go in terms of research and application in cancer treatment.

Bibliometric was defined by Pritchard (1969) as “the application of mathematical and statistical methods to books and other media of communication” and by Hawkins (2001) as “the quantitative analysis of the bibliographic features of a body of the literature” (Kokol et al., 2021). Currently, researchers in various fields have started utilizing bibliometrics to obtain a quick understanding of research frontiers and hotspots in specific domains. Unfortunately, there has been no bibliometric analysis of the literature in the field of TCM in cancer. Consequently, despite the steady increase in the number of TCM-related research papers in the field of cancer, the overall development of knowledge, research hotspots, and research trends remain unclear. Therefore, in this study, we used R software, VOSviewer, and CiteSpace to analyze the relevant literature on TCM in cancer. Our goal was to explore the changes and development trends of research hotspots between cancer and TCM from 2002 to 2022 and to identify potential research hotspots that can provide a reference for future research. Looking to the future, a better understanding of the current situation and potential of TCM is crucial for the sustainable development of this field.

## 2 Materials and methods

### 2.1 Data collection

The data used in this study were retrieved and downloaded from WoSCC (Guangxi Medical University purchase edition) on 5 September 2022. We used the following search formula: [TS=(traditional Chinese medicine OR traditional medicine, Chinese OR Chinese traditional medicine OR medicine, Chinese traditional) AND TS=(cancer OR cancers OR tumor OR tumors OR neoplasm OR neoplasia OR neoplasias) AND DOP=(2002-01-01/2022-09-05) AND DT=(Article OR Review)] AND LA=(English). After removing the literature irrelevant to the study, we observed 7,129 papers (with no duplicates). The retrieved papers were saved in a plain text format and exported as full records along with their cited references.

### 2.2 Data analysis

To analyze the annual publications, Origin 2018 was used. Additionally, the bibliometrix package of R software (version 3.6.3) (version 4.0, http://www.bibliometrix.org) (Aria and Cuccurullo, 2017), VOSviewer (version 1.6.17) (van Eck and Waltman, 2010), and CiteSpace (version 6.1.4) (Chen, 2006) were employed to visually analyze data and draw scientific knowledge maps. To ensure data accuracy and reliability, two different authors conducted data extraction and analysis management, respectively.

VOSviewer was used to visualize the co-authorship network of countries/institutions, co-citation analysis of sources, and co-occurrence of keywords. In co-authorship network analysis, we set the following parameters: minimum number of documents of a country ≥5; minimum number of documents of an organization ≥30. In the co-citation of source analysis, we set the following parameters: minimum number of citations of a source ≥500. Additionally, in the co-occurrence of keyword analysis, the parameters were set as follows: minimum number of occurrences of a keyword ≥30, and we excluded “traditional Chinese medicine,” “cancer,” and “tumor” keywords. The journal impact factors (IFs) were retrieved from Journal Citation Reports (JCR) of 2021.

## 3 Results

### 3.1 General landscapes of included documents on TCM in cancer

**FIGURE 1:**
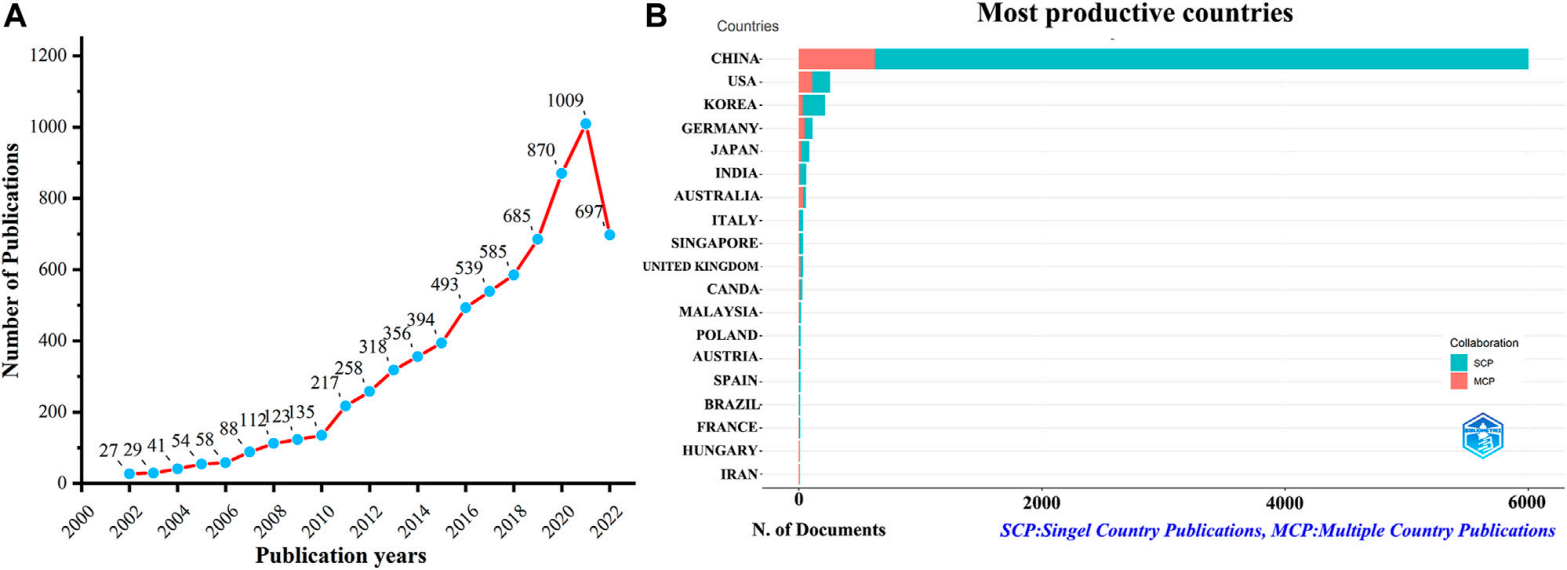
Trends in annual publication outputs in the field of TCM in cancer from 2002 to 2022. **(A)** Trends of annual publication outputs. **(B)** Distribution of corresponding authors’ countries and cooperation.

A total of 7,129 documents were collected from WoSCC without duplicates. Figure 1A shows that publications about TCM in cancer are increasing each year. From 2002 to 2010, the number of relevant documents showed a slow upward trend. From 2010 to 2021, the number of related documents increased rapidly, of which 1,009 documents were published in 2021. By 5 September 2022, 697 relevant documents had been published.

**FIGURE 2:**
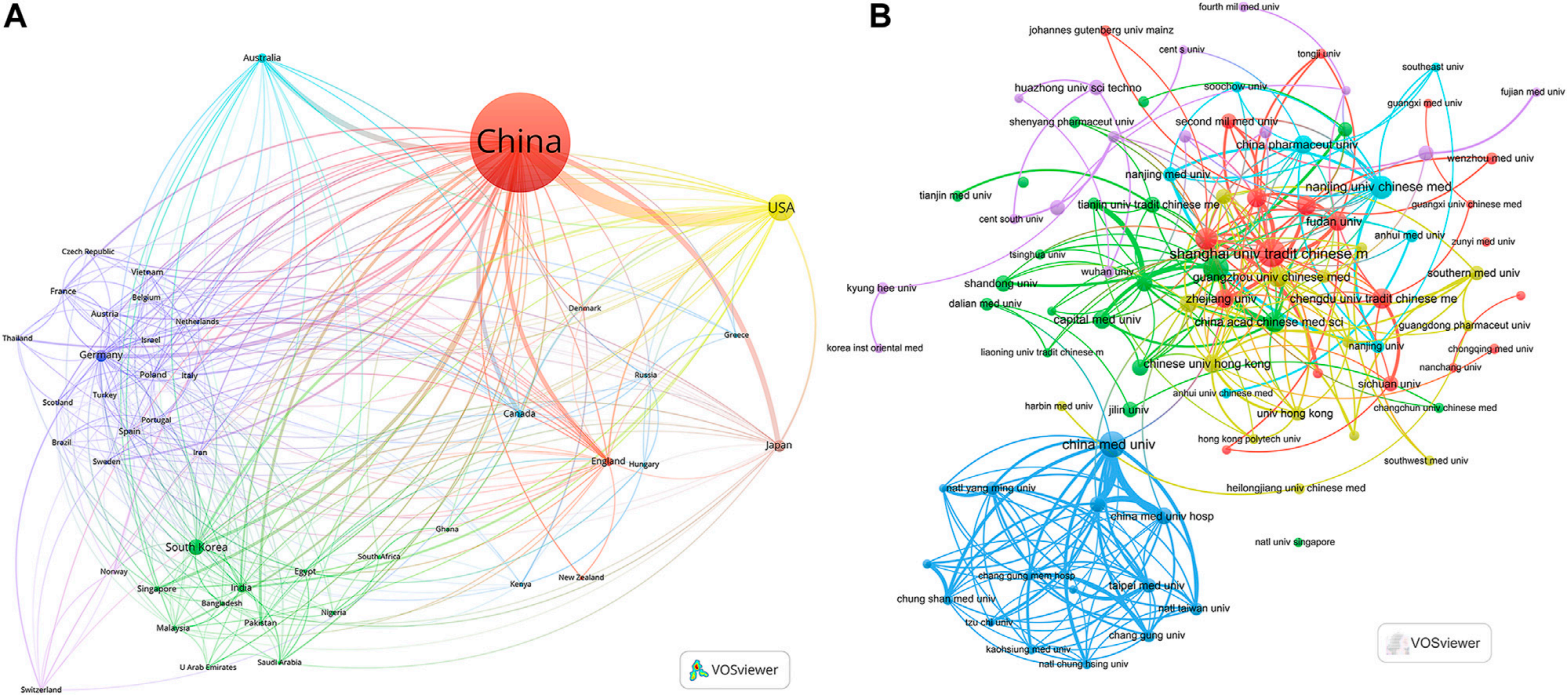
Map of countries/regions and institutions in the field of TCM in cancer from 2002 to 2022. **(A)** Map of cooperation between different countries. **(B)** Map of cooperation between different institutions.

Based on the corresponding authors’ countries, we found that China (*n* = 5,999) had been the most productive, followed by the USA (*n* = 527), Republic of Korea (*n* = 215), Germany (*n* = 111), and Japan (*n* = 85). Interestingly, we found that among the top five countries in terms of the number of published documents, only 10.50% and 15.30% from China and Republic of Korea were multiple country publications (MCPs), respectively, which is well below 43.20% and 44.10% in the United States and Germany, respectively (Figure 1B; Table 1). Meanwhile, Figure 2A indicates that China had the most extensive collaboration with other countries in the field of TCM in cancer. Additionally, the collaboration map demonstrates that China Medical University (*n* = 682) and Shanghai University of Traditional Chinese Medicine (*n* = 593) were representative centers of collaboration (Figure 2B; Table 2).

**TABLE 1:** Most relevant countries by corresponding authors of traditional Chinese medicine in cancer.

Country	Article	SCP	MCP	Freq (%)	MCP_Ratio (%)
China	5,999	5,371	628	84.10	10.50
United States	257	146	111	3.60	43.20
Republic of Korea	215	182	33	3.00	15.30
Germany	111	62	49	1.60	44.10
Japan	85	64	21	1.20	24.70
India	60	48	12	0.80	20.00
Australia	58	22	36	0.80	62.10
Italy	32	25	7	0.40	21.90
Singapore	32	21	11	0.40	34.40
United Kingdom	32	17	15	0.40	46.90
Canada	29	16	13	0.40	44.80
Malaysia	19	13	6	0.30	31.60
Poland	15	13	2	0.20	13.30
Austria	12	5	7	0.20	58.30
Spain	12	10	2	0.20	16.70
Brazil	10	8	2	0.10	20.00
France	10	6	4	0.10	40.00
Hungary	8	3	5	0.10	62.50
Iran	8	3	5	0.10	62.50

MCP, multiple-country publication; SCP, single-country publication.

**TABLE 2:** Most relevant affiliations of traditional Chinese medicine in cancer.

Affiliation	Articles (n)
China Medical University	682
Shanghai University of Traditional Chinese Medicine	593
Beijing University of Chinese Medicine	411
Nanjing University of Chinese Medicine	396
Guangzhou University of Chinese Medicine	299
The Chinese University of Hong Kong	276
Fudan University	251
Chengdu University of Traditional Chinese Medicine	245
Zhejiang Chinese Medical University	242
Shanghai Jiao Tong University	217
China Pharmaceutical University	204
Sun Yat-sen University	201
Zhejiang University	196
China Medicine University Hospital	189
Fujian University of Chinese Medicine	178

### 3.2 Journals and co-cited journals

**FIGURE 3:**
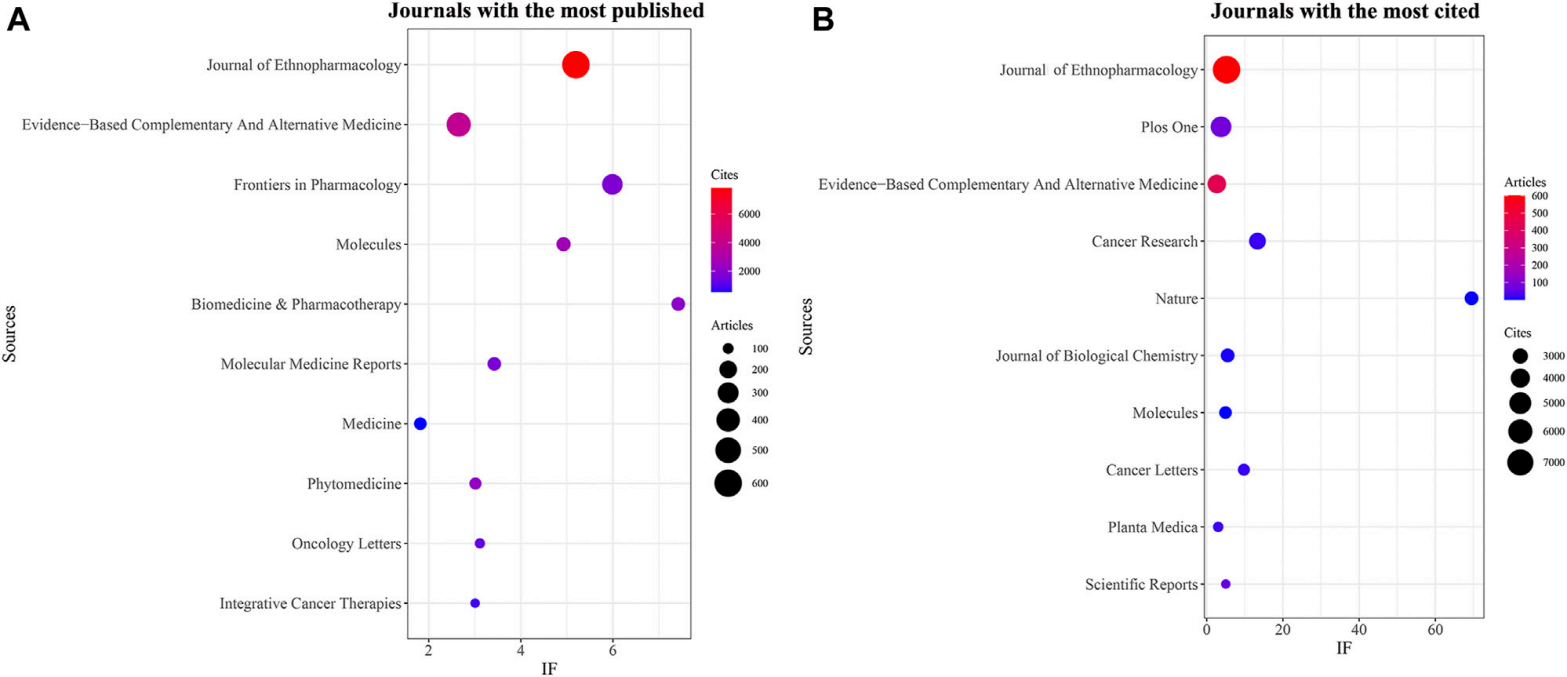
Journal with the largest number of articles published and the journal with the largest number of citations. **(A)** Journal with the largest number of articles published. **(B)** Journals with the largest number of citations.

**FIGURE 4:**
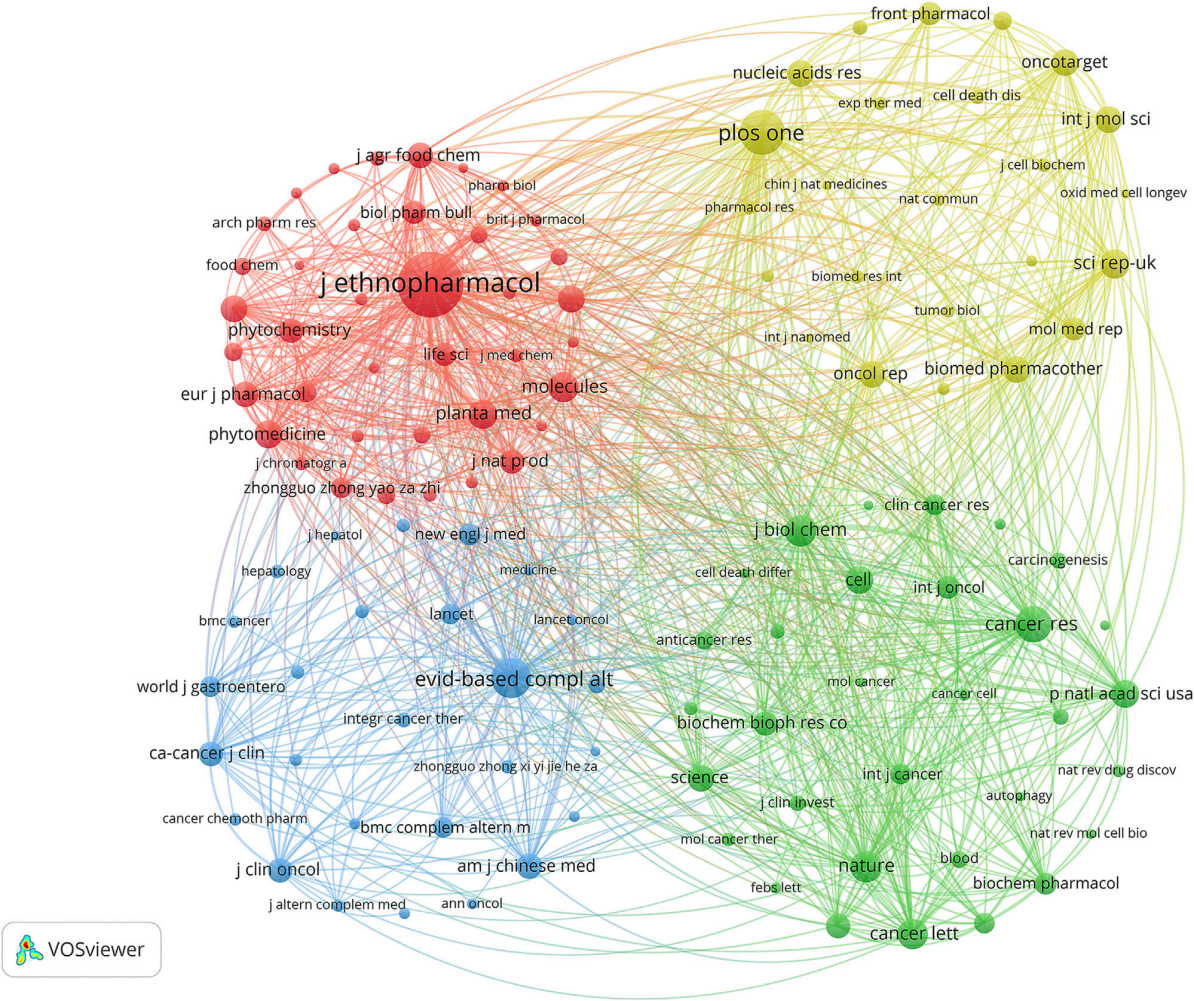
Co-cited journals involved in TCM in cancer.

The Bibliometrix and ggplot2 packages of R software (version 3.6.3) were used to analyze the journals with the most published documents and the journals with the most citations in this field. Additionally, VOSviewer (version 1.6.17) was utilized to conduct the co-cited journal analysis. The results indicated that 7,129 documents were published in 1,074 academic journals (Annex 1). Table 3; Figure 3A demonstrate that the most published documents were in the Journal of Ethnopharmacology (*n* = 602, IF = 5.195), followed by Evidence-Based Complementary and Alternative Medicine (*n* = 434, IF = 2.65), Frontiers in Pharmacology (*n* = 288, IF = 5.989), Molecules (*n* = 132, IF = 4.927), and Biomedicine and Pharmacotherapy (*n* = 125, IF = 7.149). Moreover, Table 4; Figure 3B show that the most frequently cited journals were the Journal of Ethnopharmacology (*n* = 7,831), followed by PLOS One (*n* = 4,538, IF = 3.752), Evidence-Based Complementary and Alternative Medicine (*n* = 3,825), Cancer Research (*n* = 3,340, IF = 13.312), and Nature (*n* = 2,756, IF = 69.504). The co-cited journal maps revealed that the Journal of Ethnopharmacology and Evidence-Based Complementary and Alternative Medicine were representative centers of collaboration (Figure 4). These findings suggest that the Journal of Ethnopharmacology and Evidence-Based Complementary and Alternative Medicine may be influential journals in the field of TCM in cancer. Furthermore, these findings suggest that there is a lack of publications on the achievements of TCM in cancer in top journals. This further indicates the need for improving the depth and quality of research in this area.

**TABLE 3:** Top 10 journals with the most published articles.

Journal	Documents	IF (2021)	Cites
Journal of Ethnopharmacology	602	5.195	7831
Evidence-Based Complementary and Alternative Medicine	434	2.65	3,825
Frontiers in Pharmacology	288	5.988	1763
Molecules	132	4.927	2,621
Biomedicine and Pharmacotherapy	125	7.419	2094
Molecular Medicine Reports	123	3.423	1,666
Medicine	113	1.817	528
Phytomedicine	108	6.656	2,253
Oncology Letters	98	3.11	1,268
Integrative Cancer Therapies	97	3.007	881

**TABLE 4:** Top 10 journals with the most cited journals.

Journal	Cites	IF (2021)	Documents
Journal of Ethnopharmacology	7831	5.195	602
PLOS One	4,538	3.752	79
Evidence-Based Complementary and Alternative Medicine	3,825	2.65	434
Cancer Research	3,340	13.312	21
Nature	2,756	69.504	2
Journal of Biological Chemistry	2,755	5.486	6
Molecules	2,621	4.927	1
Cancer Letters	2,558	9.756	21
Planta Medica	2,463	3.007	27
Scientific Reports	2,448	4.996	69

### 3.3 Most cited references and reference burst

We used the bibliometrix package of R software to identify the top 20 most cited references in the field of TCM and cancer (Table 5). We found that these references had been cited more than 320 times, and they came from 19 different journals, indicating that research in this area has not yet achieved significant breakthroughs. Interestingly, there were no dominant journals among the top 20 cited references. Among the top three cited references were “Antioxidant activity and phenolic compounds of 112 traditional Chinese medicinal plants associated with anticancer,” “Natural products in drug discovery,” and “Beneficial effects of green tea—A review.” However, upon closer inspection, we found that these articles only provided a general overview of the relationship between TCM and cancer.

**TABLE 5:** Top 20 cited references related to traditional Chinese medicine on cancer.

Paper	DOI	Total citation	TC per year
Cai YZ, 2004, LIFE SCI	10.1016/j.lfs. 2003.09.047	1722	90.63
Harvey AL, 2008, DRUG DISCOV TODAY	10.1016/j.drudis. 2008.07.004	1,191	79.40
Cabrera C, 2006, J AM COLL NUTR	10.1080/07315724.2006.10719518	1,147	67.47
Li S, 2013, CHIN J NAT MEDICINES	10.3724/SP.J.1009.2013.00110	738	73.80
Li-Weber M, 2009, CANCER TREAT REV	10.1016/j.ctrv. 2008.09.005	660	47.14
Lin Y, 2008, CURR CANCER DRUG TAR	10.2174/156800908786241050	603	40.20
Shishodia S, 2005, ANN NY ACAD SCI	10.1196/annals.1352.010	484	26.89
Zhang AH, 2012, ANALYST	10.1039/c1an15605e	460	41.82
Yang HJ, 2006, CANCER RES	10.1158/0008-5472.CAN-05-4529	457	26.88
Wand XH, 2007, MED RES REV	10.1002/med.20077	432	27.00
Huang Q, 2007, MED RES REV	10.1002/med.20094	420	26.25
Gong J, 2012, J CONTROL RELEASE	10.1016/j.jconrel. 2011.12.012	395	35.91
Thomford NE, 2018, INT J MOL SCI	10.3390/ijms19061578	388	77.60
Tang J, 2009, J ETHNOPHARMACOL	10.1016/j.jep. 2009.08.009	386	27.57
Efferth T, 2007, TRENDS MOL MED	10.1016/j.molmed. 2007.07.001	381	23.81
Kitano H, 2007, NAT REV DRUG DISCOV	10.1038/nrd2195	373	23.31
Kronenberg F, 2002, ANN INTERN MED	10.7326/0003-4819-137-10-200211190-00009	363	17.29
Efferth T, 2008, CLIN INFECT DIS	10.1086/591195	331	22.07
Brinker AM, 2007, PHYTOCHEMISTRY	10.1016/j.phytochem. 2006.11.029	329	20.56
Amagase H, 2011, FOOD RES INT	10.1016/j.foodres. 2011.03.027	326	27.17

Blue font indicates the year when the paper was published, and the red font indicates the journal in which the paper was published.

**FIGURE 5:**
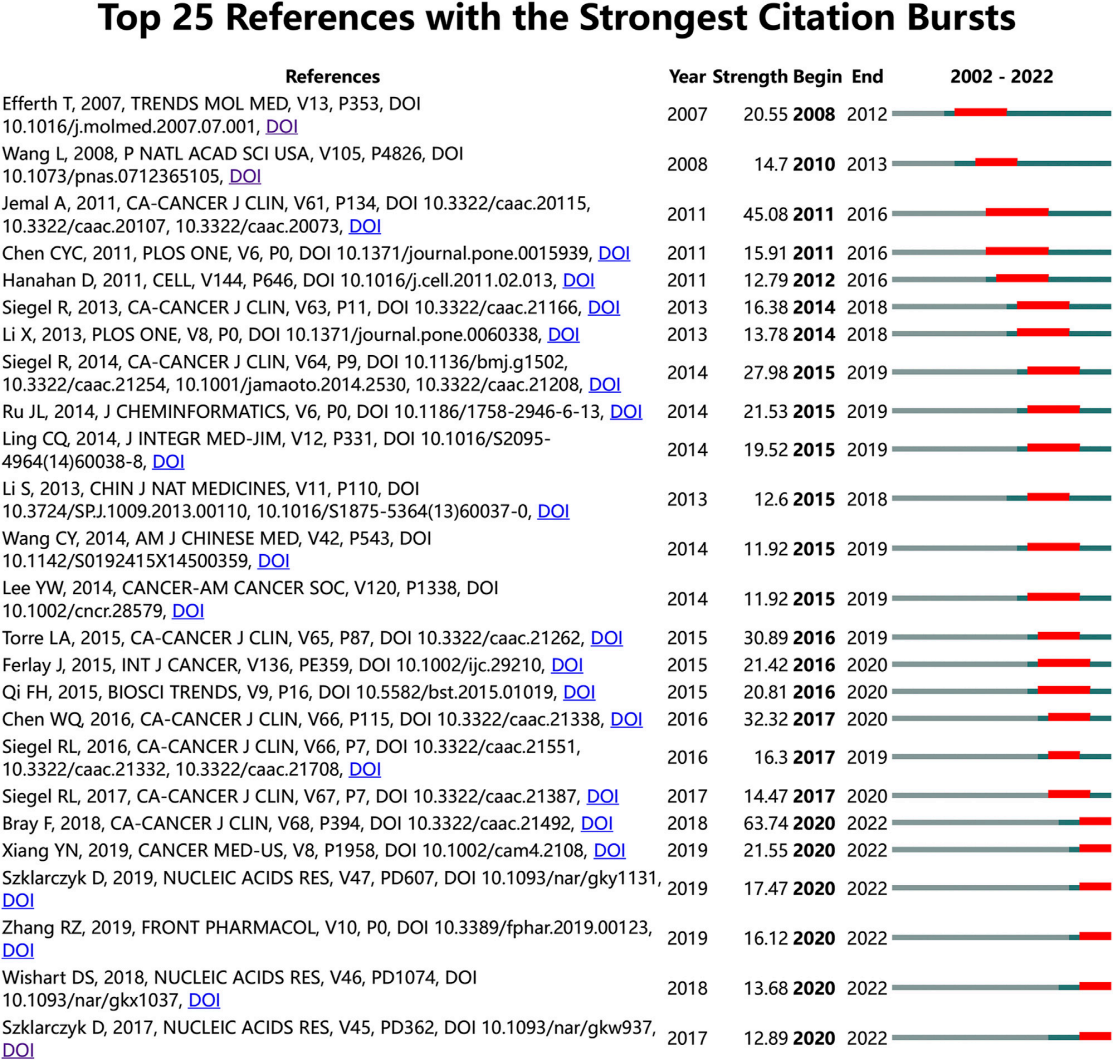
Top 25 references with the strongest citation bursts on TCM in cancer research.

To identify the most significant citation bursts for TCM in cancer, we used CiteSpace (selection criteria: top 25; the number of states: 2; and minimum duration: 2). This yielded 223 references with the strongest citation bursts, 25 of which are displayed in Figure 5. Among them, “Global cancer statistics 2018: GLOBOCAN estimates of incidence and mortality worldwide for 36 cancers in 185 countries (strength: 63.74),” “Cancer Statistics, 2010 (strength: 45.08),” and “Cancer statistics in China, 2015 (strength: 32.32)” were the top three with the most vigorous citation bursts. Interestingly, the titles of the three most cutting-edge citation bursts were “The STRING database in 2017: quality-controlled protein–protein association networks, made broadly accessible,” “DrugBank 5.0: a major update to the DrugBank database for 2018,” and “Network pharmacology databases for traditional Chinese medicine: review and assessment.” To gain further insights into the research frontiers and hotspots in the field of TCM in cancer, we corresponded the DOIs of the 25 citations in Figure 5 to the titles in Annex 2. Of these citations, “cancer reports” were the most numerous (32%), followed by network pharmacology-related literature (24%), including reviews of network pharmacology and frequently used databases in network pharmacology studies. These findings suggest that network pharmacology has become a hot topic in recent years in TCM research related to cancer. It is worth noting that from the aforementioned literature, it can be seen that network pharmacology research heavily relies on network databases. This may lead to concerns about the originality of its research findings.

### 3.4 Keyword clusters and evolution

Keyword clusters are an excellent way to understand the research hotspots and directions in a field. In this study, a total of 12,998 keywords were extracted using VOSviewer. Table 6 shows that the top 20 keywords appear more than 200 times. The most frequently occurring keywords were apoptosis (*n* = 1,147), followed by an expression (*n* = 1,080), *in vitro* (*n* = 809), activation (*n* = 731), cells (*n* = 675), inhibition (*n* = 557), growth (532), and NF-kappa B (*n* = 492).

**TABLE 6:** Top 20 keywords related to traditional Chinese medicine on cancer.

Rank	Keyword	Count
1	Apoptosis	1,147
2	Expression	1,080
3	*In vitro*	809
4	Activation	731
5	Cells	675
6	Inhibition	557
7	Growth	532
8	NF-kappa B	492
9	Proliferation	455
10	Inflammation	388
11	Pathway	376
12	Therapy	355
13	Oxidative stress	338
14	Mechanisms	332
15	Extract	286
16	Breast cancer	247
17	Induction	247
18	Metastasis	237
19	Chemotherapy	233
20	Cancer cells	223

**FIGURE 6:**
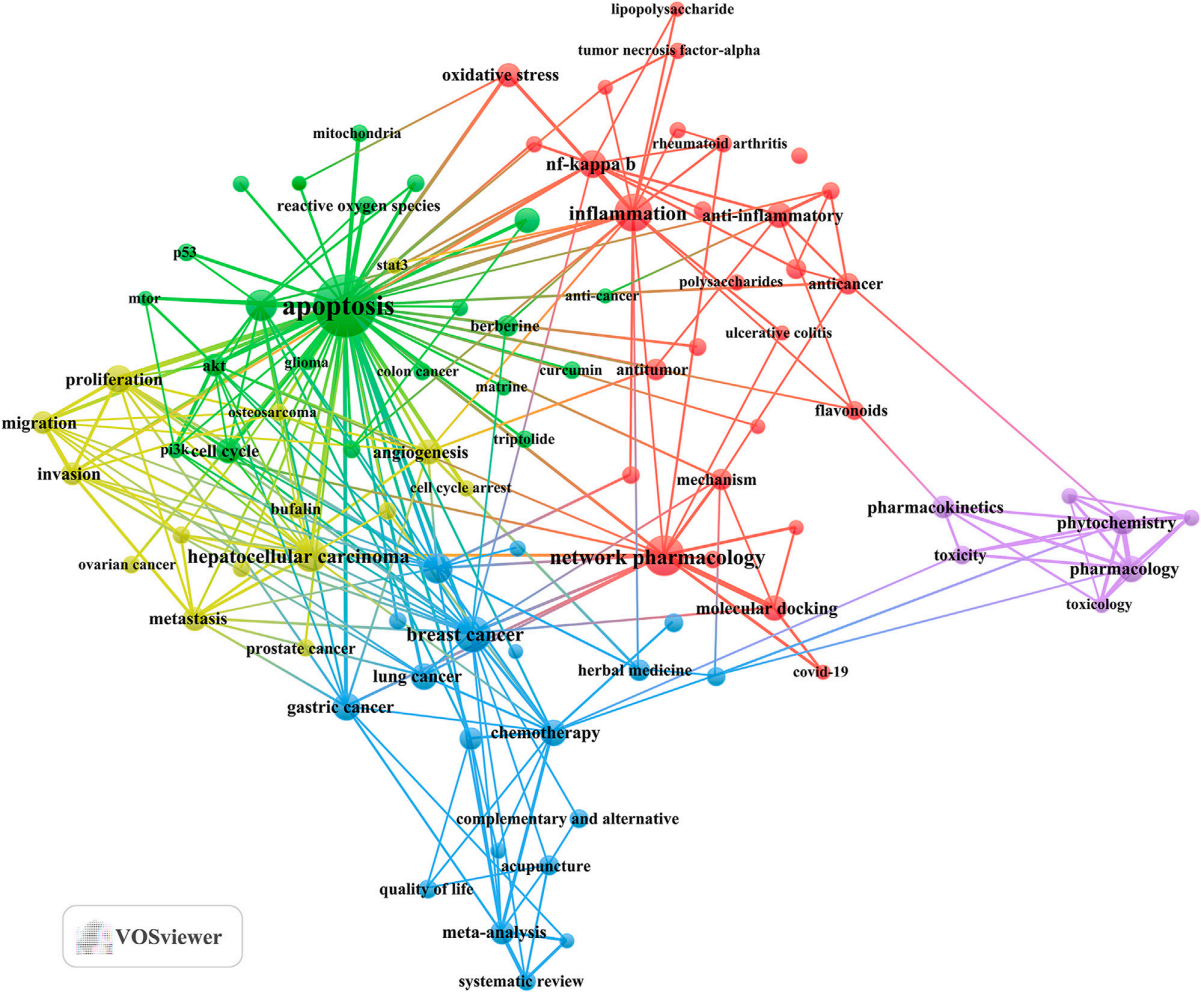
Keyword co-occurrence map of publications on TCM in cancer research.

Then, 91 keywords were selected according to the “minimum number of occurrences of a keyword ≥30” to draw the keyword cluster map (Figure 6), and a total of five clusters with different colors are observed in Figure 6. There were 28 keywords in the anti-inflammatory and experimental method cluster (red dot), including network pharmacology, inflammation, anti-inflamm, NF-kappa B, and oxidative stress. There were 22 keywords in the cancer type and molecular mechanism cluster (green dot), including breast cancer, lung cancer, gastric cancer, colorectal cancer, and chemotherapy. There were 19 keywords in the cancer type and quality control cluster (blue dot), including breast cancer, lung cancer, gastric cancer, colorectal cancer, and chemotherapy. There were 15 keywords in the cancer type and malignant characteristic cluster (yellow dot), including angiogenesis, bufalin, cell cycle arrest, epithelial–mesenchymal transition, and hepatocellular carcinoma. There were seven keywords in the pharmacology and toxicology cluster (purple dot), including pharmacokinetics, pharmacology, phytochemistry, quality control, toxicity, and toxicology (Annex 3).

**FIGURE 7:**
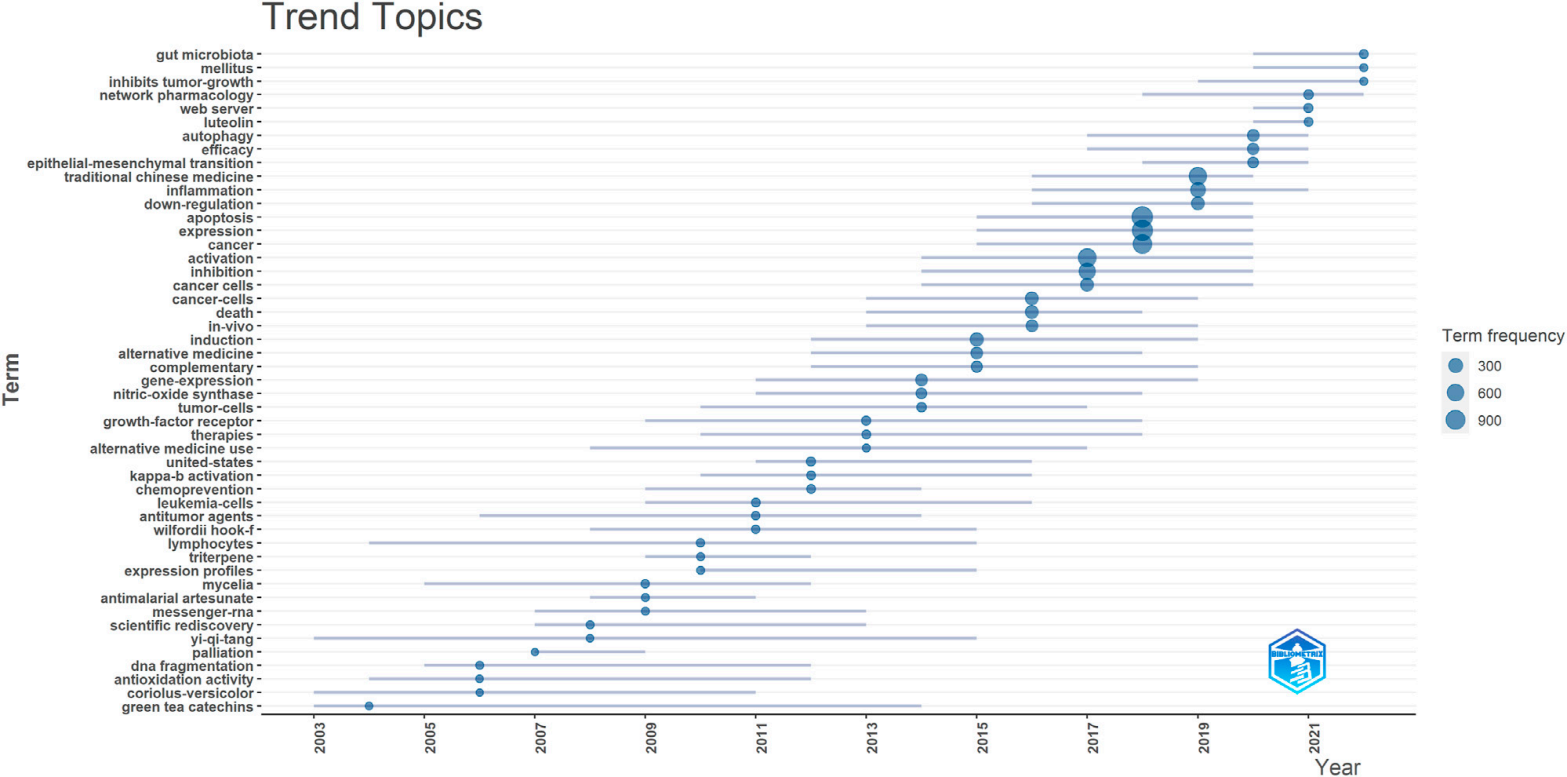
Trend topics on TCM in cancer research.

In addition, we utilized the bibliometrix package of R software to generate a trend topic map (Figure 7). The trend topic map is a useful tool for identifying the chronological progression of specific research themes within a given field, enabling us to examine the evolution of that field over time. By analyzing the trend topic map presented in Figure 7, we were able to identify the research focus and evolution track of each stage of TCM in cancer research. Our findings indicate that current research in this field is centered on gut microbiota and network pharmacology.

In general, through keyword clusters and evolution, we found that the research hotspots of TCM in cancer mainly focus on cancer types, molecular mechanisms, and research methods. Of these, molecular mechanisms primarily involve anti-inflammatory and apoptotic pathways. Investigations rely on network pharmacology and analysis of gut microbiota.

## 4 Discussion

### 4.1 General information

To gain a better understanding of the research focus and direction of TCM in cancer, we conducted a bibliometric and visual analysis. Our study included a total of 7,129 papers spanning the period from 2002 to 2022. Our findings showed an upward trend in the number of papers on TCM in cancer research over the last 20 years, with a significant growth trend in the past 5 years. This study indicates that TCM in cancer research is increasingly favored by researchers.

In the research field of TCM in cancer, China had published the highest number of papers (5,999). Furthermore, all of the top 15 affiliation publishing papers were from China, with China Medical University having the largest number of published papers (682). This result was not surprising, as China has unique advantages in TCM research, particularly in the area of cancer. It is worth noting that the United States was the second country to publish papers in this field, followed by the Republic of Korea and Japan, which have a deep cultural influence from China. This indicates that TCM in cancer has received worldwide attention from researchers.

A total of 7,129 papers were published in 1,074 journals, with the leading journals including the Journal of Ethnopharmacology, Evidence-Based Complementary and Alternative Medicine, Frontiers in Pharmacology, Molecules, and Biomedicine, and Pharmacotherapy. Interestingly, the Journal of Ethnopharmacology and Evidence-Based Complementary were also among the most cited journals. This suggests that these journals are key publications in the field of TCM in cancer research.

### 4.2 Hotspots and development trends

Through the analysis of the most cited references, reference bursts, keyword clusters, and keyword trend topics, we identified the research hotspots and frontiers of TCM in cancer. Our study revealed three points that are worth paying attention to in the field of TCM in cancer.

The first is the hotspots and trends of TCM in cancer types. TCM preparations, either alone or in combination with chemotherapeutic agents, have been widely used in cancer treatments (Lu et al., 2021). Our keyword cluster analysis revealed that “breast cancer,” “colorectal cancer,” “gastric cancer,” “liver cancer,” and “lung cancer” were the most researched cancer types in this field. In fact, many studies have confirmed the effectiveness of TCM in treating these five types of cancer and have elucidated their mechanisms of action. For instance, Wang et al. (2022) reported that dandelion extract inhibited triple-negative breast cancer cell proliferation by interfering with glycerophospholipids and unsaturated fatty acid metabolism. Zhai et al. (2022) found that red ginseng polysaccharides exhibited anti-ferroptosis effects in the lung and breast cancer cells by targeting GPX4. Additionally, Pien Tze Huang was found to inhibit colorectal cancer growth and immune evasion by reducing beta-catenin transcriptional activity and PD-L1 expression (Chen et al., 2022). Moreover, Ouyang et al. (2023) showed that Chinese dragon’s blood ethyl acetate extract suppressed gastric cancer progression through the induction of apoptosis and autophagy mediated by the activation of MAPK and downregulation of the mTOR-Beclin1 signaling pathway. These studies suggest that “breast cancer,” “colorectal cancer,” “gastric cancer,” “liver cancer,” and “lung cancer” are the current hotspots and trends in TCM for cancer treatment. While TCM preparations have shown promise in cancer treatment, it is important to note that their efficacy and safety must be rigorously evaluated through well-designed clinical trials. The lack of standardization and quality control of TCM products can pose challenges in conducting such trials, as variations in the composition and dosage of herbs can affect their therapeutic effects and increase the risk of adverse reactions. Additionally, the potential interactions between TCM and conventional cancer treatments must be carefully considered, as some herbs may interfere with the pharmacokinetics and pharmacodynamics of chemotherapy drugs, potentially leading to treatment failure or toxicity.

Second, the hotspots and trends of the molecular mechanisms of TCM in cancer include anti-inflammatory and apoptosis pathways. Keyword and citation analysis revealed that the anti-cancer mechanisms of TCM mainly focus on inducing apoptosis, anti-inflammation, regulating oxidative stress, and gut microbiota. Apoptosis or programmed cell death is a critical process for the normal functioning of cells in the body. In cancer, however, the abnormal regulation of apoptosis can lead to uncontrolled cell growth and survival, which can contribute to tumor progression. TCM has been shown to induce apoptosis in cancer cells through various mechanisms, such as activating pro-apoptotic proteins or inhibiting anti-apoptotic proteins (Xu et al., 2019; Shen et al., 2022). Additionally, inflammation and oxidative stress are also important processes in cancer development and progression. There is substantial evidence showing that TCM has anti-inflammatory and anti-oxidant effects, which are manifested through the regulated expression of inflammatory factors and oxidative stress (Xie et al., 2020; Wang J. et al., 2022). Notably, keyword cluster analysis revealed high occurrence frequencies of “NF-kappa B,” “MAPK,” “Akt,” “mTOR,” “p53,” and “pi3k.” These results suggest that TCM may exert apoptosis-inducing, anti-inflammatory, and anti-oxidative mechanisms by regulating pathways such as NF-kappa B, MAPK, PI3K/Akt, and mTOR (Qian et al., 2018; Wang TF. et al., 2022; Vidal et al., 2022; Zhang et al., 2022; Zhou et al., 2022). Furthermore, gut microbiota has gained more attention in TCM anti-cancer research. Gut microbiota is a complex micro-ecosystem that is referred to as the second genome of the human body (Chen et al., 2021). Therefore, regulating gut microbiota can help in the treatment of cancer patients. Currently, the anti-cancer mechanisms of TCM by interfering with gut microbiota mainly include two aspects: regulating the dysbiosis and metabolism of gut microbiota. Shao et al. (2021) concluded that Xiao-Chai-Hu-Tang inhibited tumor progression by reversing gut dysbiosis, particularly by reducing the abundance of Parabacteroides, Blautia, and Ruminococcaceae bacterium. Wang et al. (2021) demonstrated that evodiamine could inhibit the development of colitis-associated cancer by regulating gut microbiota metabolites, especially tryptophan metabolism. These studies suggest that “apoptosis,” “anti-inflammation,” “oxidative stress,” and “gut microbiota” are hotspots and trends of the anti-cancer mechanisms of TCM.

Third, hotspots and trends in research methods for TCM in cancer were identified. Based on the analysis of the most cited references, reference burst, keyword clusters, and keyword trend topics, network pharmacology was found to be a hotspot and trend in research methods for TCM in cancer. The concept of network pharmacology was put forward by Hopkins (2007) as a comprehensive discipline that integrates system biology, information networking, computer science, and pharmacology. By exploring the interaction between biological functions and diseases at each node of the network, network pharmacology coincides with the holistic view of TCM, making it a popular method in the research of the pharmacological basis and mechanism of TCM in cancer (Li et al., 2017). In addition, network pharmacology has the advantage of providing a systematic approach to studying the complex interactions between TCM and the human body. Moreover, the keyword cluster analysis showed that “molecular docking” and “metabolomics” were highly correlated with network pharmacology. Several studies combined metabolomics with network pharmacology to explore the mechanism of TCM in treating cancer. For instance, Qi et al. (2021) found that ketenone may inhibit A549 cells through MMP9, STAT3, and TYMS, indirectly affecting the pathway of pyrimidine metabolism, pantothenic acid, and CoA biosynthesis. Thus, metabolomics combined with network pharmacology is conducive to understanding the anti-cancer mechanism of TCM. Therefore, the research method of metabolomics combined with network pharmacology is a hotspot and trend in research methods for TCM in cancer. Nevertheless, it is imperative to acknowledge that network pharmacology is solely a prognostic tool for identifying targets. The pharmacological efficacy of drugs necessitates rigorous experimental substantiation.

### 4.3 Strengths and limitations

This research may help researchers better understand this field and explore new directions. However, some limitations must be addressed. First, we only used the WoSCC database as the source of data, which may have resulted in the omission of some publications. Nevertheless, the WoS database is a high-quality digital literature database recognized by researchers and widely regarded as the most suitable database for bibliometric analysis (Thelwall, 2008; Merigo and Yang, 2017; Cheng et al., 2021). Therefore, our data source selection is reliable. Second, we only analyzed publications in English, which could lead to source bias. Third, our study did not analyze authors, as there are numerous Chinese authors with the same name, and a large proportion of TCM researchers are Chinese. Therefore, author analysis would be misleading.

In general, despite these limitations, our research provides a comprehensive overview of the general situation, hotspots, and research trends in this field.

## 5 Conclusion

Our study clearly showed the main research hotspots and frontiers of TCM in cancer research. The following is a summary of the knowledge points and research hotspots in the field of TCM in cancer:

a. TCM in cancer research has garnered attention from researchers worldwide, with the United States, Republic of Korea, Germany, and Japan being the most active countries. Extensive cooperation among countries has been carried out.
b. The Journal of Ethnopharmacology and Evidence-Based Complementary and Alternative Medicine are the most active journals that publish TCM in cancer-related documents. The Journal of Ethnopharmacology is also the most cited journal, indicating that it is a representative journal in the TCM in the cancer research field.
c. Breast cancer, colorectal cancer, gastric cancer, liver cancer, and lung cancer are the hotspots and trends of TCM in cancer types.
d. Pathway-related “apoptosis,” “anti-inflammation,” and “oxidative stress” are the hotspots and trends of the anti-cancer mechanism of TCM. Additionally, “gut microbiota” is a hotspot worth paying attention to.
e. The research method of metabolomics combined with network pharmacology is a hotspot and trend of research methods for TCM in cancer.

In conclusion, our study offers valuable insights into the research trends and hotspots within the field of TCM in cancer. These findings can aid researchers in gaining a comprehensive understanding of this domain and facilitate the exploration of new avenues for future investigations. By identifying the current research landscape and potential areas of focus, our study equips researchers with the necessary knowledge to navigate this field more effectively and pursue innovative directions in their studies.

## Data Availability

The raw data supporting the conclusion of this article will be made available by the authors, without undue reservation.
