# Supplementary material for: Bibliometric and visual analysis in the field of traditional Chinese medicine in cancer from 2002 to 2022
Source: Front Pharmacol. 2023 Jul 4;14:1164425. doi: 10.3389/fphar.2023.1164425 (PMC10352617; doi:10.3389/fphar.2023.1164425)
Supplement: Supplementary file 2 [file Table3.DOCX]

Cluster 1 (28 items)

anti-inflamm

ationanti-inflammatory

anti-tumor

anti-inflamm

ationanti-inflammatory

anti-tumor

antitumor activity

covid-19

cytokines

flavonoids

gut microbiota

inflammation

lipopolysaccharide

mapk

mechanism

metabolomics

molecular docking

molecular mechanism

network pharmacology

nf-kappa b

nuclear factor-kappa b

oxidative stress

polysaccharide

polysaccharides

rheumatoid arthritis

tnf-alpha

tumor necrosis factor

ulcerative colitis

Cluster 2 (22 items)

akt

anti-cancer

apoptosis

autophagy

berberine

celastrol

cell cycle

cervical cancer

cisplatin

colon cancer

curcumin

cytotoxicity

glioma

matrine

mitochondria

mtor

p53

pi3k

reactive oxygen species

ros

tanshinone iia

triptolide

Cluster 3 (19 items)

acupuncture

breast cancer

chemotherapy

colorectal cancer

complementary and alternative medicine

drug resistance

gastric cancer

herbal medicine

liver cancer

lung cancer

meta-analysis

natural products

non-small cell lung cancer cancer

protocol

quality of life

randomized controlled trial

review

systematic review

tumor microenvironment

Cluster 4 (15 items)

angiogenesis

bufalin

cell cycle arrest

epithelial-mesenchymal transition

hepatocellular carcinoma

invasion

metastasis

migration

multidrug resistance

osteosarcoma

ovarian cancer

pancreatic cancer

proliferation

prostate cancer

Cluster 5 (7 items)

pharmacokinetics

pharmacology

phytochemistry

quality control

toxicity

toxicology

traditional uses
